# Clathrin-mediated entry and cellular localization of chlorotoxin in human glioma

**DOI:** 10.1186/1475-2867-11-27

**Published:** 2011-08-12

**Authors:** Marzenna Wiranowska, Lucrecia O Colina, Joseph O Johnson

**Affiliations:** 1 Department Pathology & Cell Biology, College of Medicine, University of South Florida, Tampa FL, USA; 2 Analytic Microscopy Core Facility, H. Lee Moffitt Cancer Center and Research Institute, Tampa FL, USA

## Abstract

**Background:**

Chlorotoxin (TM601), a scorpion venom- derived 36-AA peptide, is an experimental drug against recurrent glioma with tumor specificity but unknown route of intracellular distribution. The aim of this study was to evaluate the route of entry and cellular localization of TM601 in glioma cells.

**Results:**

We have found that in human gliomas, lung carcinoma and normal vascular endothelial cells, TM601 localizes near trans-Golgi while in normal human dermal fibroblasts (NHDF) and astrocytes it is dispersed in the cytoplasm. The uptake of TM601 by U373 glioma cells is rapid, concentration and time dependent, not affected by inhibitors such as filipin (caveolae-dependent endocytosis) and amiloride (non-selective macropinocytosis), but significantly affected by chlorpromazine (clathrin-dependent intracellular transport of coated pits) resulting in intracellular build-up of the drug and clathrin near the Golgi. In contrast, TM601 uptake by NHDF cells was significantly affected by amiloride indicating that macropinocytosis is the dominant uptake route of TM601 in these cells.

**Conclusions:**

In conclusion, we found a distinct cellular localization pattern and uptake of TM601 by glioma cells differing from that found in normal cells. Further insight into the cellular processing of TM601 should assist in the development of effective anti-glioma therapeutic modalities.

## Background

TM601 is a pure, chemically synthesized chlorotoxin of 36 amino acids which was first purified from the venom of the scorpion *Leiurus quinquestriatus*. For clinical development, chlorotoxin has been manufactured using solid phase chemical syntheses, and it is called TM601. It is known that TM601 has a similar homology and structure as other venom peptides and in that resembles members of the family of small disulfide-rich proteins characterized by a knotted topology [1]. It was first found to be an inhibitor of small conductance chloride channels [2]. Both inhibition of invasion and inhibition of metalloproteinase-2 (MMP-2) activity have been previously observed in glioma cells treated with chlorotoxin [3,4]. Recently, a similar *in vitro *finding was reported for human umbilical vein endothelial cells (HUVEC) treated with TM601 [5]. TM601 is not cytostatic or cytotoxic to tumor or vascular endothelial cells *in vitro*. However, potent pleiotropic anti-angiogenic effects of TM601 *in vivo *were reported. These effects include a decreased tumor growth in chick chorioallantoic membrane assay as well as decreased intra-tumoral hemoglobin levels. In addition, it was shown that TM601 injected intravenously *in vivo *in mice significantly decreased new blood vessel growth [5].

Recently, the *in vitro *and *in vivo *tumor targeting properties of the peptide have been shown to be retained following conjugation to a near-infra red fluorescent dye [6], nanoparticles [7,8], and polymers [9]. Similar observations were made in *in vivo *animal models using chlorotoxin conjugated to ^131^Iodine-(^131^I -) [10]. The nanoparticles with multiple chlorotoxin molecules on their surface have shown enhanced inhibition of invasion *in vitro *compared to monomeric chlorotoxin [11]. The tumor targeting properties of TM601 conjugated to ^131^I -administered intravenously were evaluated in Phase I clinical studies for solid tumors [12] and locally in Phase II clinical studies of recurrent glioblastoma multiforme [13]. These studies showed that after the local delivery of ^131^I-TM601 to the resection cavity, the radiolabeled protein complex was detectable at the tumor cavity site for several days [13,14].

TM601 does not bind to non-transformed cells such as human neurons, astrocytes and fibroblasts as well as to over 15 normal human tissues [5,15]. However, using histochemical methods, TM601 binds selectively to glioma cells and other tumors of neuroectodermal origin [15]. Furthermore, recently, annexin A2 was identified as a binding partner for TM601 in multiple human tumor cell lines and normal human endothelial cells HUVEC [16]. Despite its known specificity for tumor cells including glioma, the exact route of entry, intracellular trafficking, and cellular localization of TM601 in glioma cells compared to control normal cells has not yet been resolved. In this study we evaluated TM601 cellular entry by three different endocytic pathways. We used chlorpromazine, an inhibitor of clathrin-mediated intracellular transport of clathrin-coated pits, filipin, an inhibitor of cholesterol-dependent caveolar cell transport, and amiloride, an inhibitor of Na+/H+ exchange in ligand-independent, non-selective transport by macropinocytosis.

The amiloride-sensitive, ligand-independent macropinocytosis involves indiscriminant sampling of large extracellular volumes by large endocytic vesicles, macropinosomes. However, both filipin and chlorpromazine affect formation of small endocytic vesicles containing involuted specific plasma membrane domains such as the cholesterol rich domain of caveolae or other domains of receptors. Therefore, the activities of filipin and chlorpromazine substantially differ from that of amiloride by having a selective effect on ligand-dependent endocytosis [17-21].

Here we provide evidence that TM601 enters glioma cells mainly via a clathrin-dependent chlorpromazine- sensitive pathway leading to the Golgi, whereas in normal fibroblasts the predominant transport mechanism for TM601 is macropinocytosis. In addition, we show that the cellular localization and processing of TM601 in glioma cells differs profoundly from that found in normal astrocytes and fibroblasts but is similar to normal human endothelial cells.

## Results

### Time and Dose Response and Cellular Localization of TM601

**Figure 1:**
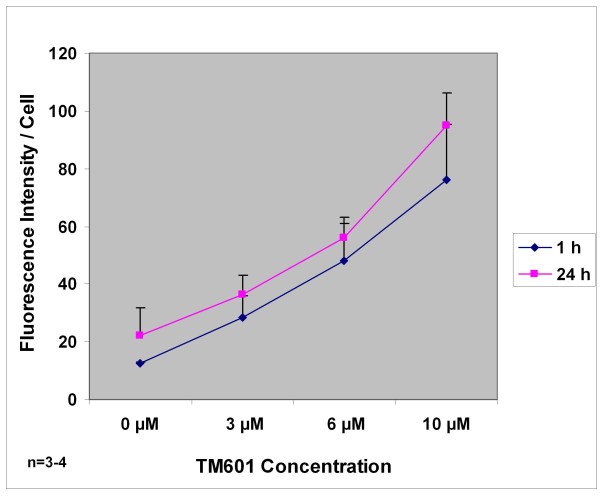
**TM601 Time and Dose Response in U373 glioma cells**. TM601 dose response of U373 glioma incubated with 0, 3, 6, and 10 μM TM601 for 1 h or 24 h and evaluated by immunocytochemistry and confocal microscopy in n = 3-4 experiments. The fluorescence intensity/cell was obtained studying 50-100 cells/each concentration of TM601 (at 1 h and 24 h) in each experiment. Presented are means and SD for each TM601 concentration.

To asses TM601 time and dose response, human glioma U373 cells were incubated with 0, 3, 6, and 10 μM TM601 for 1 h or 24 h, and followed by immunocytochemistry and confocal microscopy (Figure 1). TM601 fluorescence intensity of glioma cells showed that TM601 entered cells in a dose-dependent (Figure 1) manner showing a linear increase in cell's fluorescence intensity with increased concentrations of TM601 at 1 h and 24 h incubation times. This increase was also observed at 25 and 50 μM concentrations of TM601. The levels of TM601 were similar at 1 h and 24 h at any given concentration (Figure 1). When additional earlier time points, e.g., 10 and 30 minutes were tested using 10 μM TM601 concentrations, we observed that TM601 achieved its maximum cellular level and plateaued within first 30 minutes of incubation. The uptake of TM601 by U373 glioma was rapid and occurred only under physiological (37°C, 5% CO_2_) conditions. There was no TM601 detected in the cells when they were either incubated at 4°C, or at 37°C, in the absence of 5% CO_2._

### TM601 Cellular Localization: Immunocytochemistry

**Figure 2:**
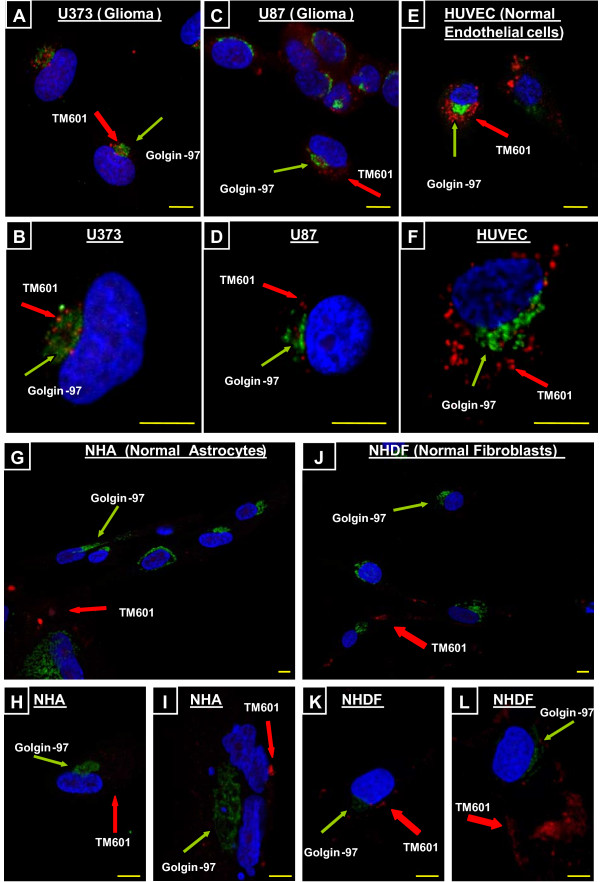
**Cellular localization of TM601 and Golgin-97 by immunocytochemistry**. Immunocytochemistry followed by confocal microscopy showed cellular localization of TM601 (red) in relation to Golgin-97 (green) and nucleus (blue) in human cells: U373 glioma (A, B), U87 glioma (C, D), normal umbilical vascular endothelial HUVEC (E, F), normal astrocytes NHA (G, H, I) and normal fibroblast NHDF (J, K, L) cells. The figure shows a similar Golgi localization of TM601 in gliomas (U373, U87) and HUVEC cells but in NHA and NHDF cells, TM601 is seen away from the trans-Golgi. Shown are data representative of n = 8 experiments. The scale bars on the confocal images = 10 μm, magnification 1890 × (A, C, E, H, I, K, L), 4410 × (B, D, F), 630 × (G, J).

The confocal evaluation of human glioma cells (U373 and U87) treated for 24 h with 10 μM TM601, detected TM601 near trans-Golgi (Figure 2A, B, C, D). Golgin-97, a 97 kDa member of the granin protein family is a peripheral membrane protein localized on the cytoplasmic face of the Golgi apparatus and it is unique to the Golgi apparatus of most vertebrate species. Therefore, Golgin-97 was used as a marker for the identification of the Golgi apparatus in the glioma and normal cells. Similar to glioma cells, normal human umbilical vascular endothelial (HUVEC) cells showed TM601 localization near the trans-Golgi membranes (Figure 2E, F). However, following the same treatment of normal human dermal fibroblasts (NHDF) or normal human astrocytes (NHA) using 10 μM TM601 for 24 h, TM601 appeared as diffused staining in the cytoplasm, distant from the trans-Golgi area (Figure 2G, H, I, J, K, L). Therefore, TM601 cellular localization in these two normal cell types, NHA and NHDF, differed profoundly from what was observed in glioma cells and normal HUVEC cells. The negative non-TM601 treated cell controls showed no staining.

### TM601 Cellular Localization: Direct Fluorescence

**Figure 3:**
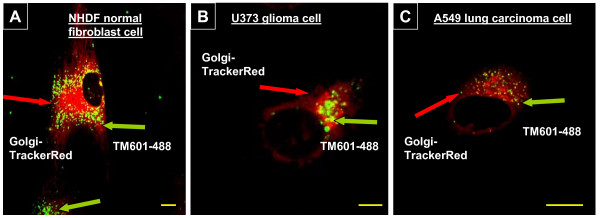
**Cellular localization of TM601-488 and Golgi complex-TX Red (Golgi-Tracker) by direct fluorescence**. Images of live cells and cellular localization of TM601-488 (green) in relation to the Golgi-complex-Texas-Red (red) detected by direct fluorescence in NHDF normal human fibroblasts (A), U373 glioma (B), and A549 lung carcinoma (C). The figure shows similar perinuclear localization in the Golgi area of TM601-488 in glioma U373 and lung carcinoma A549, but in NHDF cells, TM601-488 is seen in the cytoplasm away from the Golgi area and outside the cell membrane. The data are representative of n = 9 experiments. Scale bars = 10 μm, magnification 1260 × (A), 630 × (B), 2000 × (C).

A similar cellular localization and a granular staining of TM601 as seen using immunocytochemistry, were also observed in live cells using a direct fluorescence evaluation of 24 h Alexa-488 conjugated TM601 (TM601-488) treated cells followed by the treatment with the BODIPY TR C5-ceremide sphingolipids complex to BSA (Golgi complex-Texas (TX) Red -Golgi tracker). The negative non-TM601 treated cell controls showed no staining. The evaluated tumor cell lines such as human glioma U373, human lung carcinoma A549 (Figure 3B, C) and normal HUVEC cells (data not included) showed perinuclear localization of TM601 in the Golgi area confirming the previous observation by immunocytochemistry. Again, in the normal human NHDF cells TM601-488 was detected in the cytoplasm away from the Golgi area (Figure 3A).

**Figure 4:**
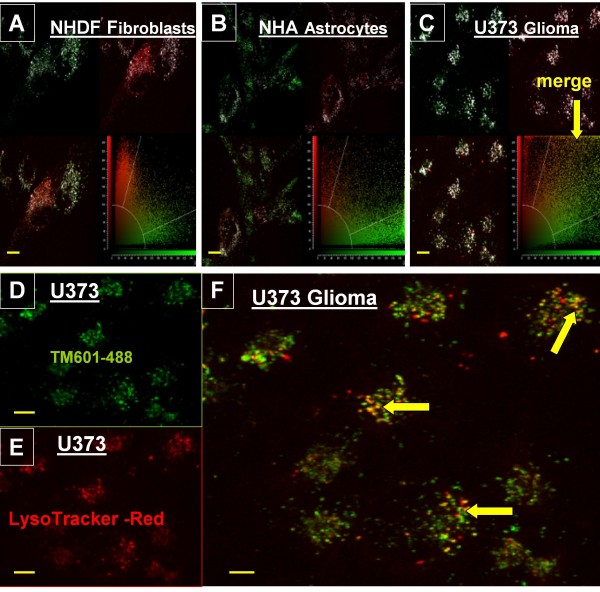
**Cellular colocalization of TM601-488 and Lyso-Tracker-Red DND 99 by direct fluorescence**. Images of live cells and cellular colocalization of TM601-488 (green) and Lyso-Tracker-Red DND 99 (red) detected by direct fluorescence in NHDF normal human fibroblasts (A), NHA normal human astrocytes (B), and U373 human glioma (C) using the Colocalization Tool (as described in the Methods). An overlay mask (white pixels) placed on each image visualizes the colocalization of pixels above the background threshold showing the highest level of white pixels in the U373 glioma (C). The following three fluorescent color channels were used: green (TM601-488), red (Lyso-Tracker- Red), and yellow (merge of red and green fluorescence) to determine the extent of colocalization of TM601 and LysoTracker by confocal microscopy. The merge red and green fluorescence in glioma cells (F) was compared to the controls of TM601-488 (D) alone or LysoTracker Red (E) alone. The images in D, E, and F are representative of n = 7 experiments. The Colocalization Tool in A, B, C was used in one out of 7 experiments evaluating total of 14-29 cells in 3 fields. Scale bars = 10 μm, magnification 630 ×.

Further evaluation of a granular TM601 localization in the Golgi area in glioma cells, involved studies of acidic organelles such as lysosomes and/or late endosomes often positioned near the Golgi apparatus and the nucleus. The human glioma U373 cells were compared with normal human NHDF and NHA cells following the treatment with TM601-488 and Lyso-Tracker-Red DND 99 and evaluation of direct fluorescence. The colocalization of TM601 and LysoTracker was higher in human glioma cells when compared to both normal human astrocytes and fibroblasts showing merged red and green fluorescence (Figure 4). This finding indicated a higher level of TM601 in lysosomes of glioma cells than in normal NHA and NHDF cells suggesting that there may be different TM601 endocytic pathways in glioma versus normal fibroblasts and astrocytes. To further study this difference between TM601 uptake by U373 glioma and normal NHDF cells we used three different inhibitors of endocytosis: filipin, chlorpromazine and amiloride.

### Endocytosis of TM601: Studies with Inhibitors

#### Filipin

**Figure 5:**
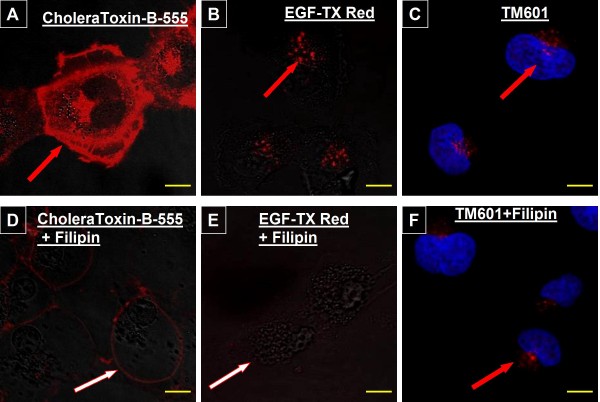
**Effect of Filipin on uptake of TM601, CholeraToxin and EGF by glioma cells**. Images of live U373 glioma cells treated with 1 μg/ml CholeraToxin B-555 (A, D), or 1 μg/ml EGF-TX-Red (B, E), or immunocytochemistry of U373 glioma treated with 10 μM TM601 (C, F) in presence or absence of filipin (5 μg/ml). There was no visible effect of filipin treatment on the level or pattern of staining for TM601, but EGF and cholera toxin staining was affected. Data are representative of total n = 4 - 5 experiments. Scale bars = 10 μm, magnification 1890 ×.

Human U373 glioma cells (Figure 5A, B, C, D, E, F) and normal NHDF fibroblasts (data not shown) treated with or without filipin followed by either TM601-488 or TM601 at 10 μM concentration for 1 h or 24 h were evaluated by confocal microscopy following either immunocytochemistry (Figure 5C, F) or direct fluorescence (Figure 5A, B, D, E). Both approaches did not show a detectable effect of filipin on the level of fluorescence of TM601 treated cells. However, when U373 glioma cells were evaluated using positive controls of epidermal growth factor (EGF) or cholera toxin uptake (known to be affected by filipin treatment [17,22]), the inhibitory effect of filipin on the level of EGF and cholera toxin staining was observed as expected (Figure 5A, B, D, E).

#### Chlorpromazine and Amiloride

##### Glioma cells

**Figure 6:**
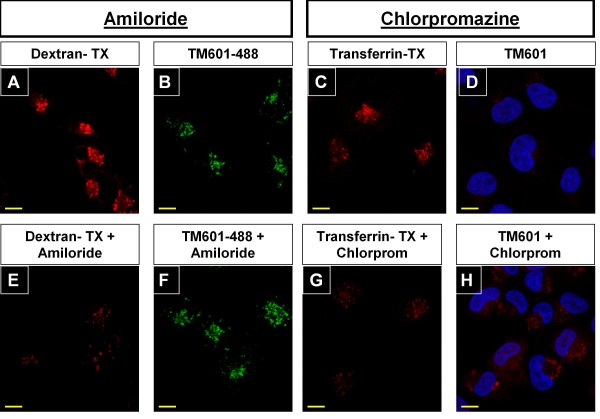
**Effect of chlorpromazine and amiloride on uptake of TM601, dextran and transferrin by glioma cells**. Images of U373 glioma cells (treated with: dextran-TX at 1 mg/ml (A), dextran-TX at 1 mg/ml in presence of amiloride 300 μM (E), TM601-488 10 μM (B), TM601-488 10 μM in presence of amiloride 300 μM (F), transferrin-TX at 35 μg/ml (C), transferrin-TX at 35 μg/ml in presence of chlorpromazine 10 μg/ml (G), TM601 10 μM (D) and TM601 10 μM in presence of chlorpromazine 10 μg/ml (H). Direct fluorescence is shown in A, B, C, E, F, G and immunocytochemistry in D, H. The level of staining of both controls, dextran and transferrin, were decreased by amiloride or chlorpromazine, respectively. Data are representative of total n = 3 experiments. Scale bars = 10 μm, magnification 1890 ×.

**Figure 7:**
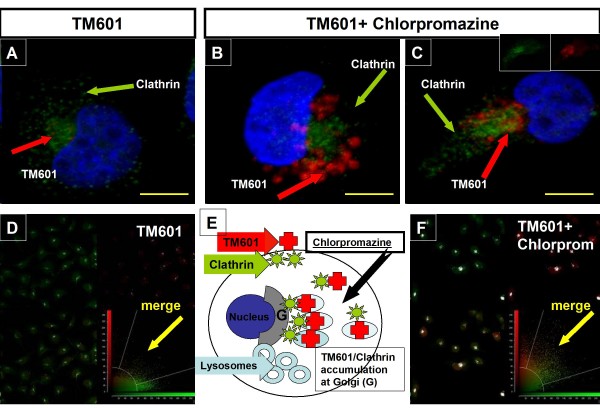
**Cellular colocalization of TM601 and clathrin light chain in chlorpromazine treated U373 glioma cells**. Immunocytochemistry of U373 glioma cells followed by confocal microscopy showed cellular localization of TM601 (red) and clathrin (green) in relation to the nucleus (blue) in TM601 treated control (A) and in TM601 and 10 μg/ml chlorpromazine treated cells (B, C). The chlorpromazine treatment resulted in accumulation of TM601 in the perinuclear region (B, C). The schematic illustration of chlorpromazine action on TM601 uptake is shown (E). The cellular colocalization of TM601 (red) and clathrin light chain (green) was detected by immunofluorescence using the Colocalization Tool (D and F) as described in the Methods. An overlay mask (white pixels) placed on each image visualizes the colocalization of pixels above the background threshold (D and F). It shows that more of white pixels were observed in TM601 and 10 μg/ml chlorpromazine treated (F) than in TM601 (D) only treated glioma cells. This is due to a higher level of TM601 in the presence of chlorpromazine resulting in higher red immunofluorescence which merged with green immunofluorescence derived from clathrin. The following three fluorescent color channels were used: red (TM601), green (clathrin light chain), and yellow (merge of red and green fluorescence) to determine the extent of colocalization of TM601 and clathrin light chain by confocal microscopy. The merged red and green fluorescence in glioma cells (C) was compared to the controls of TM601 (red insert in C) alone or clathrin light chain (green insert in C) alone. Data are representative of total n = 3 experiments. The Colocalization Tool in D and F was used in one out of 3 experiments evaluating total of 15-26 cells in 3 fields. Scale bars = 10 μm, magnification 3780 ×.

**Figure 8:**
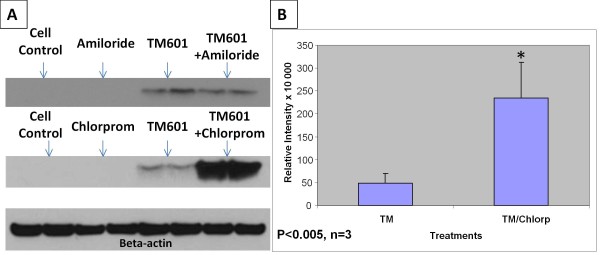
**Effect of chlorpromazine or amiloride on TM601 uptake by U373 glioma cells**. Western blots of TM601 (10 μM) - treated U373 glioma cells in presence or absence of 300 μM amiloride or 10 μg/ml chlorpromazine (A). Lysates of trypsinized cells were used at 20-25 μg protein. Beta-actin was used as a sample loading control (representative data of n = 3). Chlorpromazine (Chlorp) affected the uptake of TM601 (TM) (A, B) while amiloride had no effect on TM601 level in glioma cells (A). The statistically significant 5 fold (p < 0.005) effect of chlorpromazine is shown on the graph (B). The data represent n = 3 experiments using duplicate samples/per experiment (total of 6 samples).

Treatment of U373 glioma cells with TM601-488 or TM601 at 10 μM concentration in presence or absence of chlorpromazine 10 μg/ml, an inhibitor of clathrin-mediated transport, for 1 h (data not shown) or 24 h (Figures 6, 7, 8) affected the intracellular transport of TM601 as evaluated by direct fluorescence (data not shown), immunocytochemistry (Figures 6D, H, 7) or Western blotting (Figure 8). Controls treated with chlorpromazine alone at 10 μg/ml showed no toxicity. When U373 glioma cells were evaluated using transferrin-Texas (TX) Red as a positive control [23], the inhibitory effect of chlorpromazine on the level of transferrin staining was observed as expected (Figure 6C, G). Dual immunocytochemistry with anti-TM601 and anti-clathrin light chain (LC) antibodies showed that in TM601 treated controls clathrin was detected as a wide-spread punctate staining in the cytoplasm and at the perinuclear region, while TM601 was observed only at the perinuclear region (Figure 7A). However, in the combined chlorpromazine and TM601 treated cells, both clathrin and TM601 appeared to accumulate at the perinuclear region (Figure 7B, C). This was expected based on the known chlorpromazine effect on the clathrin-mediated intracellular transport of proteins resulting in their accumulation near the Golgi (Figure 7E). The cellular colocalization of TM601 (red) and clathrin light chain (green) was detected by immunofluorescence in U373 glioma cells using the Colocalization Tool as shown in Figure 7D and 7F.

Cells treated with TM601 and chlorpromazine showed a 5 fold significant (p < 0.005) enhancement of TM601 levels when compared to the TM601 treated controls as seen on the Western blot (Figure 8A, B). This finding confirmed the data obtained from immunocytochemistry. A similar study using a combined treatment of 10 μM TM601 or TM601-488 and 300 μM amiloride, an inhibitor of ligand -independent and non-selective transport by macropinocytosis, had no effect on TM601 cellular transport in glioma cells seen by direct fluorescence (Figure 6B, F) or on Western blot (Figure 8A). Western blot data were consistent when cell lysates were prepared from either trypsinized (Figure 8) or scraped cells (data not shown). However, when U373 glioma cells were treated with dextran-Texas (TX) Red as a positive control the inhibitory effect of amiloride on the level of dextran staining was observed as expected [21] (Figure 6A, E).

##### Normal fibroblast cells

**Figure 9:**
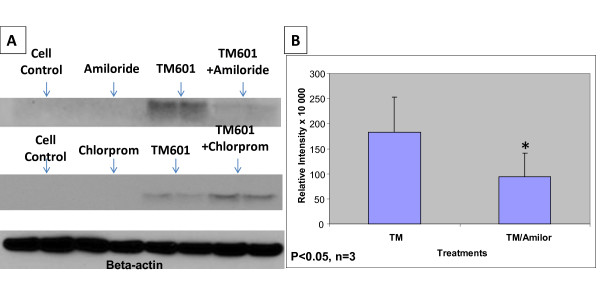
**Effect of chlorpromazine or amiloride on TM601 uptake by NHDF fibroblasts**. Western blots of TM601 (10 μM) - treated NHDF fibroblasts in presence or absence of 300 μM amiloride or 10 μg/ml chlorpromazine (A). Lysates of trypsinized cells were used at 9-12 μg protein. Beta-actin was used as a sample loading control (representative data of n = 3). The statistically significant 2 fold (p < 0.05) effect of amiloride (Amilor) on TM601 (TM) level is shown on the graph (B). The chlorpromazine had a small, statistically not significant effect on TM601 level (A). The data represent n = 3 experiments using duplicate samples/per experiment (total of 6 samples).

Normal NHDF cells incubated with 10 μM TM601-488 for 24 h in presence of 300 μM amiloride, showed decreased fluorescence by confocal microscopy. Lysates of normal NHDF cells incubated with 10 μM TM601 for 24 h in presence of 300 μM amiloride showed a 2 fold significant (p < 0.05) decrease in TM601 levels by Western blotting when compared to TM601 treated controls (Figure 9A, B). Controls treated with amiloride alone at 300 μM concentrations showed no toxicity. A similar study using a combined treatment of 10 μM TM601 and chlorpromazine at 10 μg/ml showed only a small and not significant effect (Figure 9A). These observations were consistent whether cell lysates were prepared from trypsinized (Figure 9) or scraped cells (data not shown).

## Discussion

In this study we provide data showing the cellular uptake, trafficking and localization of TM601 in glioma cells. We found that TM601 uptake by glioma cells resembles that of normal umbilical vascular endothelial cells, but is different from the one seen in normal astrocytes and fibroblasts. We have observed repeatedly, that TM601 enters glioma cell rapidly within a few minutes, and localizes in the perinuclear Golgi region, under strict physiological conditions, 37°C, 5% CO_2_, typically required for the fast (1-2.5 minutes) clathrin-mediated transport [24-26]. Clathrin-mediated endocytosis involves the concentration of high-affinity trans-membrane receptors and receptor- bound ligands into "coated pits" and adaptor protein-2 (AP-2) mediated clathrin binding to coated pits, followed by their maturation, fission and formation of clathrin-coated vesicles (CCVs). These are the major carriers for protein and lipid cargo at the plasma membrane, the trans-Golgi network (TGN), and the endosomal system [18,27]. Clathrin, a triskelion structure, is composed of three copies of clathrin heavy and up to three clathrin light chains (LC) critical for the trafficking between TGN and the endosomal system [28].

When evaluating the intracellulr trafficking of TM601 in glioma cells we focused on the clathrin LC and used dual staining for clathrin LC and TM601. While we found clathrin molecules widespread as a punctate staining most likely being part of clathrin-coated vesicles, the larger concentration of clathrin LC molecules was together with TM601 in the Golgi and adjacent perinuclear region. The accumulation of clathrin and TM601 in the perinuclear region of glioma cells may be the result of TM601 transport by clathrin-coated vesicles to the TGN.

Interestingly, we found a similar perinuclear, trans-Golgi localization of TM601 in normal human umbilical vascular endothelial cells, which was profoundly different from normal human fibroblasts and astrocytes in which TM601 was localized away from the trans-Golgi area. Our findings agree with two recent reports, showing that TM601 affects certain tumor type cells (including glioma) and vascular endothelial cells in a similar way (binds to annexin A2 and inhibits cell migration), but not the other normal human cells [5,16]. Therefore, a similar endocytotic pathway for the receptor-TM601 complex may exist in tumor cells and vascular cells.

To further evaluate TM601 localization in glioma cells we directed our focus towards acidic organelles such as lysosomes and/or late endosomes found in the perinuclear region near the Golgi. Using dual staining of live cells with fluorescent TM601 and Lysotrackers, previously used by others in studies of cellular internalization [29] we observed a higher colocalization of TM601 and Lysotrackers in glioma cells than in normal astrocytes as well as fibroblasts. This finding supports previous [15,16] and current observations that binding, uptake and cellular processing of TM601 in glioma cells all differ from those observed in normal astrocytes and fibroblasts.

To evaluate which endocytic pathway is utilized by TM601 in glioma we used three inhibitors: filipin, chlorpromazine and amiloride known to interfere with the specific stages of three different endocytic pathways and compared their effect on the level of TM601 in glioma using fibroblasts as normal controls. Filipin, a sterol-binding agent disrupts and damages structure and function of caveolae, a sub-type of lipid raft sensitive to cholesterol depletion [17,25]. It also inhibits caveolae-mediated endocytosis involving one of the types of cholesterol and sphingolipid-rich microdomains. Filipin showed no visible effect on uptake of TM601 by glioma or fibroblast cells when compared to the positive controls as measured by immunocytochemistry or direct fluorescence. As positive controls we used cholera toxin with caveolae binding specificity (gangliosides of lipid raft) and EGF ligand protein of a similar molecular size as TM601 which can be internalized through the caveolar or raft/caveolar pathway [22,27,30]. While filipin, which specifically interferes with the cholesterol-dependent caveolar transport, showed an inhibitory effect on internalization of cholera toxin and EGF in our studies, it did not affect TM601 staining intensity or pattern.

However, the possibility of filipin having some effect on TM601 uptake cannot be completely excluded since it was shown previously that inhibition of glioma matrigel invasion by recombinant chlorotoxin His-Cltx may be mediated by a filipin sensitive mechanism [3]. The reduction in matrigel invasion by His-Cltx was postulated to be at least in part mediated by filipin, and possibly involving the effect of filipin on internalization of His-Cltx bound to MMP-2 by caveoli. It is possible that TM601, unlike His-Cltx, binds to a target localized in a different region of the lipid rafts that is not sensitive to filipin. Finally, there may also be a difference between His-Cltx, and TM601 in their capacity to bind molecules such a MMP-2 believed to be associated with the cholesterol-rich areas of the lipid rafts. Therefore, the full elucidation of the effect of filipin on TM601 endocytosis requires further investigation.

To study the clathrin-dependent endocytic pathway of TM601 cellular uptake we used chlorpromazine which belongs to the cationic amphiphilic class (CAD) and phenothiazine class of drugs for mental disorders and is an inhibitor of the clathrin-coated pit -pathway. Phenothiazines were reported to inhibit clathrin-dependent endocytosis at the step of progression from type 2 coated pits to type 3 coated pits, [20]. As reported previously, chlorpromazine does not affect the initial uncoating of coated vesicles and therefore, allows cells to internalize cholera toxin [27], an observation also true for TM601. However, chlorpromazine is known to cause a loss of coated pits and associated receptors from the surface of the cell, and the accumulation of clathrin and AP-2 in the endosomal compartment [27,31-33]. Combined TM601 and chlorpromazine led to the accumulation of clathrin and TM601 in the perinuclear region corresponding to the endosomal compartment as well as significantly enhanced TM601 levels on Western blots in glioma cells. However, in normal fibroblasts (selected as a normal cell control for this study) this effect was less pronounced. As shown previously [27], the accumulation of coated endosomes following CAD treatment disrupts further processing of cholera toxin to the Golgi apparatus as observed also by us for TM601. Using the Colocalization Tool we observed that immunofluorescence of clathrin light chain (green) and TM601 (red) showed overlap at the Golgi. The observed overlap was much more pronounced in cells treated with combined chlorpromazine and TM601 than with TM601 alone, because of accumulation of TM601 (red) levels at the Golgi. The colocalization of clathrin light chain with TM601 indicates that TM601 is internalized in clathrin coated vesicles during endocytosis. Future studies are planned to further evaluate and confirm clathrin-mediated endocytosis of TM601 in glioma cells by inhibiting this pathway using specific siRNAs against clathrin heavy chain and vectors encoding dominant negative mutant forms of dynamin or Epsin.

While chlorpromazine showed only a small and not significant effect on uptake of TM601 by fibroblasts, amiloride, an inhibitor of Na+/H+ exchange in ligand-independent, non-selective transport by macropinocytosis, had a significant inhibitory effect on TM601 uptake. In contrast, amiloride had no effect on TM601 uptake by glioma cells. Macropinocytosis which occurs in many cells accompanies the signal-stimulated membrane ruffling resulting in formation of actin-driven membrane protrusions. The protrusions collapse and fuse with the plasma membrane forming large endocytic vesicles, macropinosomes, which can sample large volumes of extracellular milieu [19]. Caveolae-mediated or clathrin mediated endocytic pathways differ substantially from macropinocytosis because they result in the formation of smaller vesicles containing involuted selective plasma-membrane receptor domains. Based on the studies described above, a different endocytic pathway appears to be involved in the internalization of TM601 into glioma cells than normal fibroblasts. The TM601 receptor mediated endocytosis was observed for glioma cells while a non-receptor mediated pathway dominated in fibroblasts. In addition, we found that the uptake of TM601 into fibroblasts and normal astrocytes may be rapidly followed by a TM601 release as observed on confocal images. Therefore, levels of TM601 in fibroblasts and astrocytes may vary from cell to cell at a given point in time. The cellular localization of TM601 was similar in normal human fibroblasts and normal human astrocytes, leading us to believe that a similar endocytic pathway may be responsible for TM601 uptake in these two types of normal human cells.

As observed previously by Veiseh et al [6] tumor cell specific binding was substantially higher using chlorotoxin conjugated with Cy5.5 than with unconjugated molecule suggesting that the Cy5.5 moiety may improve tumor-specific targeting. We have made a similar observation with Alexa-488 conjugated TM601 and the monomeric molecule. We observed that TM601-488 fluorescence persists much longer than that of the monomeric TM601. Our data in glioma cells showing TM601 being taken up by clathrin-mediated endocytosis for processing into lysosomes at the endosomal compartment suggests that inducing structural changes to monomeric TM601 molecule by conjugation with Alexa- 488 or Cy5.5 or nanoparticles may affect its endocytic pathway and lysosomal degradation, protecting its activity and extending its retention in the glioma cells.

## Conclusions

In summary, our observations showed a distinct cellular localization pattern and uptake of TM601 by glioma cells differing from that found in normal cells. This finding may assist in further development of experimental strategies leading to identification of additional cellular target molecules for TM601.

## Methods

### Cells

Three human tumor cell lines: human glioblastoma grade III astrocytoma U373-MG, human glioblastoma, astrocytoma U87-MG, and human lung carcinoma A549 (American Type Culture Collection [ATCC; Manassas, VA) and three normal human cell lines: normal human dermal fibroblasts (NHDF) and normal human astrocytes (NHA) both from (Cambrex Bio Science Walkersville, Inc, Baltimore, MD), and normal human umbilical vein endothelial (HUVEC) cells (ScienCell Research Labs, San Diego, CA), were used in this study. U373, U87 and A549 cells were grown at 37°C in 5% CO_2_, in Minimum Essential Medium (MEM) supplemented with 10% heat-inactivated fetal bovine serum (FBS) and 2 mM L-glutamine, penicillin, and streptomycin, NHA cells were grown in astrocyte basic medium (ABM) (Clonogenics Medium BulletKit) supplemented with astrocyte growth supplement, 5% FBS, penicillin, and streptomycin (Lonza Walkersville, Inc. Walkersville MD), HUVEC cells were grown in vascular endothelial cell medium containing 5% FBS, penicillin, streptomycin and Endothelial Cell Growth Supplement (ScienCell).

### TM601 and TM601 antibodies

TM601 (7.6 mg/ml) was obtained from Peptisyntha, Torrence, CA. The TM601 peptide was a chemically synthesized 36-amino acid peptide containing the same amino acid sequence and four intramolecular disulfide bonds as chlorotoxin. TM601 was synthesized by solid phase chemical synthesis, refolded, and lyophilized. The peptide was purified to greater than 95% purity [5,16]. TM601-488 (1.5 mg/ml) fluorescently labeled using Alexa Fluor 488 succinimidyl ester (Invitrogen, Carlsbad, CA) was obtained from TransMolecular, Inc., (Cambridge, MA).

Anti-TM601 antibodies (1.19 mg/ml) obtained from TransMolecular, Inc., (Cambridge, MA), were raised in rabbits immunized to a conjugate between TM701 and keyhole limpet hemocyanin. Tyrosine 29 in TM601 is replaced by a phenylalanine residue in TM701. The rabbit serum from the immunized mice was purified over a protein A column, followed by an affinity purification step using a TM601-Affi-gel 10 affinity column. The specificity of the TM601 antibody was tested with synthetic TM601 using a Western blot [16].

### Direct fluorescence and dual staining

Direct fluorescence studies were performed in live cells plated (according to the manufacturer's procedure) at 10-20 × 10^3 ^cells/per insert in MatTek's glass bottom dishes (MatTek Corp. Ashland, MA). Cells were allowed to attach to the plates for 24 h using the MEM containing 10% FBS, astrocyte medium for NHA or endothelial medium for HUVEC cells. Next, the growth medium was removed and cells were either non-treated or treated for 24 h at 37°C in 5% CO_2 _with 10 μM TM601-488 in growth medium (appropriate for each cell type). Following the incubation with TM601-488, a second and/or third fluorescent label was added to detect the Golgi complex and/or lysosomes and to identify the cellular location of TM601-488. Also, negative non-TM601-488 treated cell controls were evaluated either without fluorescent label (to exclude auto fluorescence) or with fluorescent label for detection of the Golgi complex or lysosomes.

#### Staining for TM601-488 and Golgi complex

Following TM601-488 treatment, growth medium was removed and plates were washed 2 times at room-temperature with MEM without phenol red. This medium was used throughout this procedure. The BODIPY TR C5-ceremide sphingolipids complex to BSA used as a Golgi-Tracker (Invitrogen, Carlsbad, CA) was then added at 5 μM concentration for 30 min at 4°C to all plates, to detect the Golgi complex. Next, the BODIPY TR C5-ceremide-BSA was removed and cells were rinsed twice with ice-cold medium, followed by addition of room-temperature medium supplemented with 1% FBS. The plates were placed in 37°C in 5% CO_2 _incubator for 30 minutes and fluorescence immediately evaluated under confocal microscope.

#### Staining for TM601-488 and Lysosomes

Following TM601-488 treatment, media were removed and plates washed 3 times with growth medium at room temperature and Lyso-Tracker-Red DND 99 at 50 nM or LysoTracker Blue DND-22 at 50-75 nM concentration (Invitrogen, Carlsbad, CA) were added to all plates and incubated for 30 min at 37°C in 5% CO_2_. The media containing LysoTracker was then removed, fresh growth medium added, and fluorescence immediately evaluated by confocal microscopy. In n = 3 experiments a triple fluorescent labeling was used, including TM601-488, followed by the BODIPY TR C5-ceremide-BSA (as described above), and followed by LysoTracker Blue DND-22 for a simultaneous detection of TM601, the Golgi-complex and the lysosomes (data not shown).

### Immunocytochemistry

Cells were seeded onto LabTekII culture chamber-2 (CC2) 8-glass slides (Nalge Nunc International, Naperville, IL) at 20 × 10^3 ^cells/chamber in the appropriate growth medium. After 24 hours incubation at 37°C in 5% CO_2_, the growth medium was removed and replaced with fresh MEM medium with 1% FBS or with endothelial medium for endothelial cells or ABM medium for astrocyte cells. Cells were incubated with medium alone or with addition of various concentrations of TM601 (for the dose response studies) at 37°C in 5% CO_2 _for 1 hour or 24 hours. Also, an additional time points such as 10 and 30 minutes were tested as well using 10 μM TM601. Following treatment with TM601, media were removed and cells washed 2 times in phosphate buffered saline (PBS) and fixed with 4% paraformaldehyde in PBS for 15 minutes. Next, they were washed 2 times in PBS and permeabilized and blocked using blocking buffer containing 2% goat serum, 0.1% Triton X-100, 1% bovine serum albumin (BSA) in PBS for 1 hour at room temperature.

Affinity purified rabbit anti-TM601 antibody alone or in combination with mouse monoclonal anti-Golgin-97 antibody which recognize a 97 kDa protein (Invitrogen, Carlsbad, CA) or mouse monoclonal antibody to clathrin light chain (Abcam, Cambridge, MA) were diluted in blocking buffer to obtain the following concentrations 6 μg/mL (anti-TM601), 0.5 μg/mL (anti-Golgin-97), 2 μg/mL (anti-clathrin) and incubated in the chamber slides for 1 hour at room temperature. The slides were washed in 3 changes of PBS (5 minutes each) then the secondary antibody(s) diluted in blocking buffer added and incubated for 30 minutes at room temperature in the dark. The Alexa Fluor 594-labeled goat anti-rabbit IgG heavy + light (H+L) chain at 4 μg/ml (for TM601 detection) was added alone or in combination with Alexa Fluor 488-label goat anti-mouse IgG (H+L) at 2 μg/ml (Invitrogen, Carlsbad, CA, USA) for a simultaneous detection of TM601 and either Golgin-97 or clathrin- light chain. The negative non-TM601 treated cell controls were evaluated using either combination of primary and secondary antibodies or only secondary antibodies. Next, the chamber slides were washed 3 times with PBS (5 minutes each), and one time with double distilled water. The chambers were removed, slides mounted in Vectashield containing DAPI nuclear counterstain (Vector Laboratories, Inc, Burlingame, CA), cover slipped and fluorescence evaluated using confocal microscopy.

### Confocal Microscopy

Micrographs of each sample were taken with a Leica TCS SP5 laser scanning confocal microscope through a 63 ×/1.4NA or 100 ×/1.4 NA (Leica Microsystems, Germany). 405 Diode, Argon, and HeliumNeon (HeNe) 594 laser lines were applied to excite the samples and tunable filters were used to minimize crosstalk between fluorochromes. Gain, offset, and pinhole settings were identical for all samples within each experiment. The images by a differential interference contrast (DIC) microscopy were also captured using the Argon laser line. When applicable Image sections (Z- slices) at 0.5 -1 μm were captured and maximum projections were prepared with the LAS AF software version 1.6.0 build 1016 (Leica Microsystems, Germany). All images were taken at room temperature and using immersion oil. The magnification for these images is 630 × or 1000 ×. To obtain enlarged views of the cells 2 - 7 × zoom was used therefore, increasing the final magnification, e.g., 1890 × (for 3 × zoom with the 63 × lens) or 3780 × (for 6 × zoom with the 63 × lens), etc. The control plates for TM601 treated cells were used to establish the gain and offset settings to exclude non-specific secondary antibody and auto fluorescence.

#### Measurement and Quantification of TM601 Fluorescent Signal

Analysis of each cell's intensity of fluorescence for TM601 was performed using Image Pro Plus version 5.0 (Media Cybernetics, Inc., Silver Springs, Maryland). The control plates for TM601 treated cells were used to establish the "Gain" for a given light and to exclude the auto fluorescence. Individual cells were segmented using "Area of interest "(AOI) selection tool and mean intensity of TM601 signal was measured in objects 2 pixels or greater in size. Results were exported to Microsoft Excel and analyzed statistically.

#### Co-localization studies: TM601-488 and Lyso-Tracker-Red DND 99 or TM601 and Clathrin light chain

The analysis for both studies evaluating direct fluorescence (TM601-488 and LysoTracker-Red DND99) or immunofluorescence (TM601 and clathrin) was performed using the Colocalization Tool provided by Leica Application Suite (LAS) AF software version 1.6.0 build 1016 (Leica Microsystems, Germany). The resulting graphs obtained from each study represent the group of cells in the image. An overlay mask (white pixels) was placed on each image to locate colocalized pixels above the background threshold. In addition, in the colocalization study of TM601-488 and Lyso-Tracker Red DND99 the identical background thresholds, as shown on scatter plots, were set for each image to determine colocalization percentage for individual cells, which were selected with the "Region of Interest" (ROI) selection tool.

### Studies with Inhibitors of endocytosis

#### Filipin

U373 glioma and normal NHDF cells were seeded in either the LabTekII CC2 8-glass chamber slides at 20 × 10^3 ^cells/chamber (for immunocytochemistry) or in MatTek's glass bottom dishes at 20 × 10^3 ^cells/per insert (for direct fluorescence) and incubated overnight at 37°C in 5% CO_2 _in growth medium containing 10% FBS. The next day, cells were pre-treated with filipin complex from *Streptomyces filipinesis *(Sigma-Aldrich, St. Louis MO) at 1 μg/ml for 1 h at 37°C in 5% CO_2 _[34] or at 1-5 μg/ml for 15 min at 4°C [3] followed by treatment with 10 μM TM601 (for immunocytochemistry) or 10 μM TM601-488 (for direct fluorescence) for 1 h or 24 h at 37°C in 5% CO_2_. All treatments were done using the growth medium containing 1% FBS.

In addition, the effect of filipin on endocytosis of the epidermal growth factor (EGF)- biotynylated complex to Texas-Red streptavidin (TX-Red) at 1 or 10 μg/ml or CholeraToxin subunit B-555 at 1 μg/ml (both from Invitrogen, Carlsbad, CA) were evaluated. EGF was selected with a similar molecular weight (~6000 kDa) as TM601 (~4000 kDa). In these experiments cells were grown in MatTek's glass bottom dishes pre-treated with filipin at 1 or 5 μg/ml for 15 min at 4°C, followed by treatment with either EGF-TX-Red or cholera toxin-555 (in presence or absence of filipin) for 10 min at 4°C, removal of the reagents, washes and incubation of cells in growth medium containing 1% FBS for 15 min at 37°C in 5% CO_2_. The confocal evaluation of the cells' direct fluorescence was initially performed after first 10 min incubation at 4°C with the reagents (cell surface detection) and after final 15 min at 37°C incubation of cells (intracellular detection).

#### Amiloride and Chlorpromazine

The U373 glioma or normal NHDF cells were seeded in either the LabTekII CC2 8-glass chamber slides at 20 × 10^3 ^cells/chamber (for immunocytochemistry), in MatTek's glass bottom dishes at 20 × 10^3 ^cells/per insert (for direct fluorescence) or in 75 cm^2 ^flasks at 6 × 10 ^6 ^cells/flask (for Western blotting) and incubated overnight in the growth medium with 10% FBS at 37°C in 5% CO_2_. The following day, the confluent cell monolayers were pre-treated for 30 min at 37°C in 5% CO_2 _incubator with 1% FBS supplemented medium alone or with medium containing amiloride hydrochloride hydrate (Sigma-Aldrich, St. Louis MO) at 300 μM or chlorpromazine hydrochloride (Sigma-Aldrich, St. Louis MO) at 10 μg/ml. Next, the reaction medium was removed and replaced with 1% FBS supplemented growth medium containing 10 μM TM601 or 10 μM TM601-488 alone for 1 h or 24 h at 37°C in 5% CO_2_ or with addition of inhibitors (amiloride at 300 μM or chlorpromazine at 10 μg/ml) for the respective pre-treated cultures. Cell cultures were incubated for 1 h or 24 h at 37°C in 5% CO_2_. At the end of the treatment, the reaction medium was removed, cells washed 3 times and either direct fluorescence was observed (MatTek's dishes) or cells were fixed and evaluated by immunocytochemistry and confocal microscopy (chamber slides as described above); or trypsinized or scraped, cell lysates prepared and proteins evaluated by SDS/PAGE and Western blotting (tissue culture flasks).

In addition, the effect of chlorpromazine and amiloride on endocytosis of transferrin-Texas-Red (TX-Red) at 35 μg/ml according the protocol by Pho et al [23] or dextran-TX [21] at 0.1 or 1 mg/ml (both from Invitrogen, Carlsbad, CA) were evaluated, respectively. Dextran conjugate was selected with a similar molecular weight (~3000 kDa) as TM601 (~4000 kDa). In these experiments cells were grown in MatTek's glass bottom dishes. Endocytosis of transferrin was studied using confluent cell monolayers that were pre-treated for 45 min at 37°C in 5% CO_2 _incubator with 1% FBS supplemented medium alone or with medium containing chlorpromazine at 10 μg/ml. Next, the reaction medium was removed and replaced with 1% FBS supplemented growth medium containing 35 μg/ml transferrin alone or with addition of chlorpromazine at 10 μg/ml for the respective pre-treated cultures and incubated for 20 minutes at 37°C in 5% CO_2_. Endocytosis of dextran was studied also using confluent cell monolayers that were pre-treated for 30 min at 37°C in 5% CO_2 _incubator with 1% FBS supplemented medium alone or with medium containing amiloride 300 μM. Next, the reaction medium was removed and replaced with 1% FBS supplemented growth medium containing 0.1 or 1 mg/ml of dextran (21) alone or with addition of amiloride 300 μM for the respective pre-treated cultures and incubated for 1 or 24 h at 37°C in 5% CO_2_. At the end of the treatment, the reaction medium from all cultures was removed, cells washed 3 times and direct fluorescence of cells treated with transferrin -TX or dextran-TX was evaluated by confocal microscopy.

### SDS/PAGE and Western blotting

Cell monolayers treated with TM601 in presence or absence of amiloride or chlorpromazine were washed three times with PBS, trypsinized (or scraped), resuspended in ice-cold PBS, cells centrifuged and pellets washed 3 times in ice-cold PBS. Next, pellets were resuspended in ice-cold lysis buffer A containing 25 mM Tris (pH 7.6), 75 mM NaCl, 1 mM DTT, and protease inhibitor mixture (Roche Diagnostics, GmbH, Manenheim, Germany) and briefly sonicated at 4°C, centrifuged at 14 000 rpm in 4°C for 20 min, and the supernatants containing crude cytosolic fraction were collected and saved on ice. The remaining pellets were then resuspended in buffer B identical to buffer A with addition of 1% Triton X-100, and lysed in room temperature for 20 minutes. Next, the samples were centrifuged at 12 500 rpm in 4°C for 15 minutes, supernatants containing crude soluble membrane fraction were collected, combined with the first collected supernatant and protein quantified using protein assay kit and the manufacture's procedure by Pierce Biotechnology, Rockford, IL. Equal protein quantities of each supernatant (lysate) were prepared for analysis by SDS/PAGE under denaturing conditions and using DTT as a reducing agent. The level of TM601 was then determined by Western blots as follows. The NuPAGE Novex Bis-Tris Mini Gels were transferred in NuPAGE transfer buffer (Invitrogen, Carlsbad, CA) on to 0.2 μm polyvinylidene difluoride (PVDF) membrane (Invitrogen, Carlsbad, CA) and blots incubated for 1 h at room temperature in the blocking buffer containing 5% (w/v) nonfat dry milk (Carnation) in Tris-buffered saline with 0.1% Tween-20 (TBST). Next, the blots were immunoblotted in TBST blocking buffer containing either affinity-purified anti-TM601 at 2.4 μg/ml or mouse monoclonal anti-beta-actin antibodies at 0.4 μg/ml (Sigma-Aldrich, St. Louis MO, as a loading control, and incubated overnight at 4°C. The blots were washed in TBST buffer and incubated for 1 h at room temperature with goat anti-rabbit peroxidase conjugated secondary antibody at 1:20 000-1:50 000 (Jackson ImmunoResearch Laboratories, Inc. West Grove, PA) to detect TM601 or with sheep anti-mouse peroxidase conjugated anti-beta-actin at 1: 2 000 (GE Healthcare UK Limited) to detect beta-actin. After washes in TBST buffer, the blots were developed using Super Signal WestPico chemiluminescent reagent (Pierce, Rockford, IL). The intensity of TM601 bands for duplicate samples was captured using the Gel Doc XR scanner (Bio-Rad, Hercules, CA) connected to the charge-coupled device (CCD) camera for capturing image. The volume density analysis of TM601 bands was performed using Quantity One (Bio-Rad) quantitation software (version 4.6.3) subtracting the density of the pixel in the background volumes from each pixel in all sample volumes.

### Statistical analysis

Statistical analysis was performed using the Microsoft Excel Analysis Tool Pack (Redmond, WA). Descriptive statistics (mean and SD) were obtained for cell's fluorescence intensity in the studies of TM601-time and dose response (n = 3-4 experiments) evaluating 50-100 cells/per TM601 concentration at 1 h or 24 h. Also, mean and SD was obtained for the relative intensity of TM601 bands evaluated by the western blotting (n = 3 experiments) using duplicate samples. The F value for two sample variances was established and Student's t-test was used for the statistical comparison of the chlorpromazine and amiloride effect on endocytosis of TM601. Differences were considered statistically significant at p < 0.05.

## Competing interests

The authors declare that they have no competing interests.

## Authors' contributions

MW obtained the funding, designed the study, carried out the cellular treatments, participated in the immunocytochemistry assays and direct fluorescence staining, confocal microscopy, performed the statistical analysis and drafted the manuscript. LOC carried out the immunocytochemistry and western blotting, participated in confocal microscopy and assisted in evaluation and description of the data obtained from immunocytochemistry and western blotting. JOJ carried out the confocal microscopy measurement, quantification of the fluorescent signals, the evaluation and description of the data obtained from confocal microscopy and preparation of the confocal images. All authors read and approved the final manuscript.

## Authors' information

MW is an associate professor with a research interest focusing on glioma biology and treatment. She collaborated with the scientists at TransMolecular, Inc., studying cellular distribution, kinetics and localization of chlorotoxin (TM601) in glioma *in vitro *and *in vivo *in mouse models. LOC is a senior biological scientist and certified and licensed histotechnologist with an expertise in immunocytochemistry/histochemistry and western blotting. JOJ is an Analytic Microscopy Specialist and a member of Analytical Microscopy Core at H. Lee Moffitt Cancer Center with an expertise in confocal microscopy.
